# Awareness, perceived efficacy, and utilization of assisted reproductive technologies among women attending fertility clinic in a Nigerian tertiary health institution: a cross-sectional study

**DOI:** 10.11604/pamj.2022.42.181.35153

**Published:** 2022-07-06

**Authors:** Paulina Chigwara Chikeme, Chikaodili Ndidiamaka Ihudiebube-Splendor, Lilian Bekuochi Arinze

**Affiliations:** 1 Department of Nursing Sciences, Faculty of Health Sciences and Technology, University of Nigeria, Enugu Campus, Enugu, Nigeria

**Keywords:** Assisted reproductive technologies, conception, offspring, fertility clinic

## Abstract

**Introduction:**

infertility is a source of distress for couples considering the high accolade placed on having children in family settings in Nigeria. Assisted reproductive technologies (ARTs) offer a chance for parenthood to couples. However, studies on knowledge of ARTs abound in Nigeria but no previous studies exist on the extent of utilization of assisted reproductive technologies. This study thus explored the level of awareness, perceived efficacy, and utilization of ARTs among women attending fertility clinics in a Nigerian Tertiary Health Institution.

**Methods:**

this cross-sectional study which utilized a self-administered questionnaire was adopted for this study. The questionnaire contains five sections with sections A to E bordering on demographics, awareness, perceived efficacy, utilization, and factors influencing utilization respectively.

**Results:**

one hundred and seven women with infertility problems, aged between 26 and 35 years with a mean age of 31.46 ±5.72 participated in the study. Sixty-two patients (57.9%) were aware of ARTs while 97 (90.7%) believed that ART cannot address male infertility. Only 27 (25.2%) admitted to having used ART procedures before while 82 patients (76.6%) stated that the cost of the procedure was the major hindrance to use.

**Conclusion:**

awareness of ARTs is average while there is low utilization and perceived efficacy of ART. The high cost of the procedure remains the major setback to its utilization.

## Introduction

In Nigeria, like in many other countries in Africa perception of marriage and procreation are interwoven and inseparable. This, therefore, emphasizes the reason for prayers showered on newly married couples to be fruitful in their marriage. It is important to note that not all married couple ends up being fruitful as some were unable to achieve pregnancy over a period of 12 months and are therefore classified as infertile. Infertility is, therefore, the inability of a couple to conceive after regular unprotected sexual intercourse for at least one full year [1, 2]. Infertility being a global health problem is generally quoted as occurring in 8-12% of couples and is more prevalent in sub-Saharan Africa, with 10-30% of couples affected in Nigeria [3, 4]. Considering the high premium that families place on having children in Nigeria and Africa at large, being unable to get pregnant is associated with a lot of negative treatment such as deprivation, neglect, violence, psychosocial, and other marital problems [5-7]. Infertility problem is one of the commonest reasons women seek gynaecological consultation [8-11].

Infertility can be classified as primary or secondary depending on whether the woman has been pregnant before. The main causes of infertility in men and women can be hormonal, structural, or infectious in nature. In most cases, the causes of infertility could be unexplained. Also, the increasing prevalence of medical disorders such as diabetes, hypertension, hypothyroidism, and lifestyle diseases such as obesity and addictions in the young has also been implicated as the causes of infertility [12]. This is consistent with studies carried out in Nigeria which linked infertility in Nigeria to post-infectious causes such as sexually transmitted infections, puerperal sepsis, and post-abortive infection [13, 14]. Treatment of infertility issues can be either surgical or medical treatment, although, with persistent inability to conceive after these treatments, one could opt for more advanced procedures called Assisted Reproductive Technologies (ARTs) [15].

ARTs are a group of techniques that employ direct collection and in vitro processing of human gametes, as well as embryo replacement into the uterus, to overcome natural barriers to conception [16]. ART technique offers a chance at parenthood to couples who until recently would have had no hope of having a biologically related child [17, 18]. This novel innovation has today changed the human understanding of reproductive health, especially in the developed world contrary to what obtains in yet to developing countries [19]. Despite this advancement, there was found yet a low level of acceptance of ART therapeutic options by infertile couples worldwide more in developing countries with associated misconceptions, myths, and ethical and moral aftermath [9, 20, 21]. Studies on perception, awareness, and knowledge regarding ARTs therapies abound in Nigeria with conflicting reports [9, 22-26]. Despite a high degree of awareness, usage of ART services was poor in a Nigerian study [18]. Since the publication of this report, no study has been conducted to analyze the level of improvement in use, which is the purpose of this research. It is expected that the findings of this study will contribute to the literature and also bring to the limelight the level of improvement and or utilization of ARTs services based on previous reports emanating from Nigeria. It will also have implications on recommendations to improve awareness campaigns on the importance and success rate of ARTs. Hence, the specific objectives of the study were to; assess the level of awareness of ARTs services among women with fertility problems, to assess the perceived efficacy, the level of utilization, and the factors that influence the use of ARTs services.

## Methods

**Study design and participants:** a cross-sectional study was conducted between October 2020 and November 2021 among women presenting for the first time to the clinic with complaints of inability to conceive and consented to participate in the study. Women were eligible to participate irrespective of their age or duration of complaint. The study was conducted at the Tertiary Health Institution in Enugu, South-Eastern Nigeria. The hospital serves as a major referral facility for the metropolis and its environs. The infertility clinic is run once a week with an average number of 10-15 new clients seen weekly. A convenient sampling method was applied to recruit women for this study. Instruments.

**Data collection:** the data were collected through a self-developed pretested 24 item 5-sections questionnaire. Section A contained 6 items designed to collect socio-demographic information on age, marital status, religion, educational qualification, occupation, and the number of children. Section B: 4 items assessed the awareness of ARTs. In section C, 2 items assessed the perceived efficacy of ARTs, in Section D, 5 items were formulated to determine the utilization of ARTs, and in Section E, 7 items identified factors affecting the utilization of ARTs. Face and content validations of the instrument were done, and questions were rephrased where required. The reliability of the instrument was established through a pretest administered to 11 (10%) women attending infertility clinics in another institution with similar characteristics to the study facility using the test-retest statistical method and was analyzed using Cronbach´s alpha test which yielded a reliability coefficient of 0.75 confirming instrument as reliable. The data was collected by all authors and two trained research assistants during morning shifts. Before distributing the questionnaire to the women, the researchers informed them about the purpose and relevance of the study. The data collection was to ask the women to fill out a paper-based questionnaire on that spot. Beforehand, the administrative permit was obtained from the hospital management, and we worked closely with the nurses working in the clinic to plan and coordinate the data collection and we obtained maximum cooperation. Incentives were not provided for the women to fill out the questionnaires.

**Statistical analysis:** the participants´ socio-demographic, awareness, perceived efficacy, and utilization of Assisted Reproductive Technologies (ARTs) were described using descriptive statistic indicators such as frequency, percentage, standard deviation and mean. Two categories of options, “yes” or “No” was entered as response variables based on a previous study [27]. Descriptive analysis was done using SPSS software version 24.

## Results

Of the 107 women who accurately completed the questionnaires (response rate=95.5%), 52.3% (n=56) were between the ages of 26-35 years old and the mean age was 31.46 (SD =5.72) years, 94.4% (n=101) were married, 97.2% (n=104) were Christians, 98.1% (n=105) had formal education with secondary 43.0% (n= 46) and tertiary 37. 4% (n=40) education respectively, 57.9% (n=62) were self-employed, 45.8% (n=49) were childless while 41.1% (n=44) had a child (Table 1).

**Table 1:** socio-demographic profile of participants

Variables	Options	Frequency	Percentage (%)	Mean ± S.D
**Age in years**				
	19-25	12	11.2	-
	26-35	56	52.3	31.46 ± 5.72
	36-40	21	19.6	-
	Above 40 years	17	15.9	-
**Marital status**				
	Married	101	94.4	-
	Single	2	1.9	-
	Divorced	3	2.8	-
	Widowed	1	0.9	-
**Religion**				
	Christianity	104	97.2	-
	Islamism	2	1.9	-
	Pagan	1	0.9	-
	Others	-	-	-
**Educational qualification**				
	Primary	19	17.8	-
	Secondary	46	43.0	-
	Tertiary	40	37.4	-
	No formal education	2	1.9	-
**Occupation**				
	Applicant	11	10.3	-
	Civil servant	21	19.6	-
	Self-employed	62	57.9	-
	Housewife	13	12.2	-
**Number of children**				
	None	49	45.8	-
	One	44	41.1	-
	Two	12	11.2	-
	Three and above	-	-	-

On the awareness of assisted reproductive technology, out of 90.7% of the participants that have heard of ARTs, 57.9% were aware of what ART services were. Sources of information were majorly health facility (hospital) 55.7%. Majorly accepted ARTs procedure was In-vitro fertilization (IVF) 77.6%, followed by surrogacy, 67.3%, intrauterine insemination (IUI) 52.3% least was ovulation stimulation 7.5% (Table 2). On perceived efficacy of ART, the majority 90.7% believed ARTs cannot address male infertility, and 82.2% stated ARTs can fail in establishing pregnancy (Table 3).

**Table 2:** awareness of assisted reproductive technologies (ARTs) among participants

Variable	Frequency	Percentage (%)
**Have you heard of ART?**		
YES	97	90.7
NO	10	9.3
**If yes, what was your source of information (n= 97)**		
Family, relatives/friends	32	33.0
Hospital	54	55.7
Internet	8	8.2
Mass media	3	3.1
**What do you understand about ART?**		
A procedure that doctors use in educating the infertile couple	9	8.4
A procedure for stimulating ovulation in women	12	11.2
A procedure used to detect the cause of infertility in a couple	14	13.0
A procedure that involves the manipulation of sperm and egg to establish pregnancy as a treatment for infertility	62	57.9
**Which of these procedures do you know as assisted reproductive technology?**		
In-vitro fertilization (IVF)	83	77.6
Gamete intra-fallopian transfer	21	19.6
Ovulation stimulation	8	7.5
Zygote intra-fallopian transfer	16	15.0
Intrauterine insemination (IUI)	56	52.3
Surrogacy	72	67.3
Cycle tracking	27	25.2

**Table 3:** perceived efficacy of reproductive technologies (ARTs) among participants

Variables	Frequency	Percentage (%)
**Do you think ART can address male infertility?**		
Yes	10	9.3
No	97	90.7
**Do you think Assisted Reproductive Technologies can fail in establishing pregnancy in clients who use it?**		
Yes	88	82.2
No	19	17.8

On utilization of ART, 74.8% of the women have never used any ART services, out of 25.2% who used, In vitro- fertilization (IVF) 63% (n= 17) was mainly used, 77.8% (n= 21) had used it once and many 71.0% showed a willingness to use and continue the use ARTs for the treatment of infertility if given the opportunity (Table 4). Also, the majority of women 76.6% reported cost as the major hindrance to obtaining ART services, followed by fear of possible side effects 68.2%, fear of incompetence on the side of the service providers 57.0%, lack of support from spouse 53.3% and discrimination against children born through ARTs 41.1%. The last factor is religious beliefs regarding the use of artificial human fertilization 20.6% (Table 5).

**Table 4:** level of utilization of assisted reproductive technologies among participants

Variables	Frequency	Percentage
**Have you ever used any assisted reproductive technology before?**		
Yes	27	25.2
No	80	74.8
**If yes, which ART procedure did you use? (n=27)**		
In vitro fertilization (IVF)	17	63.0
Gamete intra-fallopian transfer (GIFT	1	3.7
Zygote intra-fallopian transfer (ZIFT)	1	3.7
Intrauterine Insemination (IUI)	6	22.2
Surrogacy	2	7.4
**How many times have you used ART? (n=27)**		
Once	21	77.8
Twice	4	14.8
More than twice	2	7.4
**Do you have a future intention of using ART again? (n=27)**		
Yes	18	66.7
**No**	9	33.3
**Would you use assisted reproductive technology for the treatment of infertility if given the opportunity?**		
Yes	76	71.0
No	31	29 0

**Table 5:** factors that affect the utilization of assisted reproductive technologies (ART) among participants

Variables	Yes (%)	No (%)
ART services are not affordable	82 (76.6)	25 (23.4)
Cultural and social stigmatization because ART is thought to be unnatural	37 (36.6)	70 (65.4)
Religious beliefs which are against artificial human fertilization	22 (20.6)	85 (79.4)
Fear of incompetence on the side of the service provider	61 (57.0)	46 (43.0)
Discrimination against children born through ART	44 (41.1)	63 (58.9)
Fear of possible side effects	73 (68.2)	34 (31.8)
Lack of support from a spouse	57 (53.3)	50 (46.7)

## Discussion

Making decisions requires a high level of awareness. To be well informed about a fact, one must be appropriately aware. Women attending fertility clinics in a Nigerian Tertiary Health Institution were asked about their awareness, perceived efficacy, and use of Assisted Reproductive Technologies (ARTs). ARTs had been heard about by 90.7% of the women, while ART services were known by 57.9%. Health personnel was the most common source of information (55.7%). The centre though a Tertiary health institution was not long known to treat infertility via ART services. Previously, women with infertility problems who were enlightened travelled as far as Abuja, Port Harcourt just to obtain the services, ART services even though introduced anew in this centre are yet to gain popularity as seen in older practising establishments, and couples who today visit this centre are yet to come to terms with the services, most of them are yet not regular with the appoints leading to deficiencies in in-depth knowledge. Furthermore, knowing full well that this is a unique field of expertise that necessitates specialized training, health personnel's insufficiency in imparting knowledge to consumers may be insufficient. Despite this, the proportion of awareness observed in this study was also found in a similar study in a state in Nigeria [28] and in Pakistan in which the majority of the study participants showed limited awareness about infertility management, as well as a high level of disbelief and suspicion about ART procedures [29]. In the current study, 85.6% of participants were aware of various ART treatments, with in-vitro fertilization being the most widely recognized option (IVF). In contrast to other techniques of assisted reproduction, IVF involves manually combining an egg with a sperm sample and then transferring it back into the female uterus. In comparison to other methods, it has been considered the most adaptable and successful [30]. Around 5.4 million kids were born using IVF [31] in 1978 around the world. Similarly, in Benin City Nigeria in 2011, 70.1% and 71.9% of the infertile parents described the offspring from IVF as normal and acceptable [26]. Likewise, a study titled “Experience with a comprehensive University hospital-based infertility program in Nigeria” [32] described IVF as the most acceptable choice of ARTs, widely used in developed countries of the world [33, 34]. The interest to use the IVF procedure as noted in this present study is not peculiar to Nigeria probably because of the less complexity, the method attracts more acceptance. In the midst of other methods, surrogacy was the second major chosen method thereby disproving the cultural bias or mindset of our people that a woman, who wants to be a “mother”, should deliver her baby through natural means. Surrogacy is one of the ART procedures that lawfully permits a woman to carry a pregnancy for another who will become the parent of the child. By implication, the result obtained in this study showed that cultural bias of one harbouring pregnancy for another did not in any form discourage the choice of accessing this method in this study as one would have expected among Africans [35].

The majority 89.7% knew the procedure could not address male infertility and 80.4% were aware it could fail in establishing a pregnancy. This is surprising because male factor infertility contributes either as a single factor or in a combination of female causes to bring about more than 40% of cases of infertility witnessed wide world [26, 36]. Hence, despite the level of awareness about ART services, awareness of ARTs efficacy was found very low. The high number underrating the efficacy of the procedure illustrates the direct influence that lack of sufficient knowledge and misconceptions can have on one´s attitudes [18]. This research should help healthcare professionals to educate the public about ART so that misunderstandings do not hinder public acceptance of these treatments.

The utilization of ART services was still found to be profoundly low as observed in a study of 3 years ago [33]. Twenty-five (25.2%) (n=27) of the women reported to have used IVF once, 14.8% (n=4) twice though the majority 71.0% expressed interest to make use of the technology in the future if given the opportunity based on the uncompromised benefits. This is similar to a report of the study in Ilorin [37] and Benin City [9] in Nigeria where the majority of the respondents indicated an intention to advise and share information about the existence of ARTs with friends, relatives, and neighbours. Similarly reports obtained in Anambra State [22], Sokoto [38], Northern Nigeria [33], Ibadan [39], and more still in Ilorin [18] showed low interest in to use of ART services. In the Northern part of Nigeria only a handful, 7.6% of clients were willing to embrace ART services [34] same also in Ibadan [35] and Ilorin in Nigeria study [18]. Likewise in a study in Iran [15] and Pakistan [29], IVF remains an unfamiliar and unacceptable option. That notwithstanding ART services currently are generating much interest in many parts of the world with low perception still existing in developing countries where education, poverty, and cultural background may have continued to play a great role [9]. The high cost of obtaining ART services 76.6% among others was identified as the major barrier to utilization. It is a fact in Nigeria, that government ARTs established centres are only two of the over fourteen In-vitro fertilization and Embryo transfer centres found in the country hence privately-owned ART establishments charge exorbitantly. With the majority of Nigerians living in abysmal poverty and the minimum income for only civil servants officials set at ₦18,000, which is even unpaid in certain areas, acquiring ART services remains a distant dream for the majority of infertile couples.

Depending on the service facility, an IVF cycle might cost anywhere between ₦500,000 and ₦3,500,000 [40, 41]. According to all indications, many couples struggling with infertility would have wanted this option but were unable to do so due to financial constraints. No wonder many despite the prohibitive cost of obtaining the services showed a positive attitude and willingness to go for the procedure and also promised to recommend the same to others. But dismally in Northern Nigeria [34] very few couples 7.6% showed a willingness to opt for ARTs. Their resilience could be hinged on their culture. In the Northern part of the country, polygamy is the practice in which a man is entitled to as many as possible wives and the woman could decide to abandon the initial married thus the issue of not having a child may not have any stronghold as seen in the Eastern.

Limitations: convenience sampling, like in other studies of this type, was a constraint because the ideas acquired through this study may not represent the opinions of the overall general public; The research was conducted in a specific geopolitical region of Nigeria and does not necessarily reflect the views, opinions, or awareness of ARTs in other regions of the country; infertility is a stigmatized health condition, making gathering information from these individuals difficult; there was a risk of response bias, in which the women tended to offer socially desirable responses when asked about ARTs awareness, efficacy, and use; the sample size was insufficient due to the small number of instances observed. The study should be broadened and extended to include more women who are experiencing infertility. Despite its flaws, this is an important study that should influence policy at both the district and national levels.

## Conclusion

This study explored the level of awareness, perceived efficacy, and utilization of ARTs among women attending fertility Clinics in a Nigerian Tertiary Health Institution. Our findings showed that an average number of women were aware of ARTs and major sources of their information were hospital personnel. IVF was commonly used. The majority argued the procedures could not address male infertility and many were aware it could fail in establishing a pregnancy. Utilization was profoundly low though many expressed willingness to use ART services for the treatment of infertility if given the opportunity and to encourage others to do so. The cost was the major barrier to obtaining treatment. Reducing costs of treatment and sensitizing the public about ART services will help overcome myths and misconceptions surrounding ARTs services. Nevertheless, this study was faced with some limitations such as; the relatively low number of women with fertility problems involved in the study, the exclusion of males in the study, and the involvement of only a facility within Southeast Nigeria. Further studies involving more women and men, a huge sample size, and extended health are suggested.

### What is known about this topic

- *Infertility is a global health problem and a socially destabilizing condition for couples;*
- *Assisted Reproductive Technologies (ARTs) offer a chance at parenthood to couples; who would until recently have had no hope of having “a biologically related” child;*
- *There are barriers to the effective utilization of ARTs*.

### What this study adds

- *The low use of ARTs services is comparable to that seen in other countries throughout the world;*
- *In-vitro fertilization was the most widely used ART method, whereas ovulation stimulation was the least;*
- *The exorbitant expense of the procedures is still the biggest deterrent to using ARTs services*.
